# *In Vitro* and *In Vivo* Efficacy of Albendazole Chitosan Microspheres with Intensity-Modulated Radiation Therapy in the Treatment of Spinal Echinococcosis

**DOI:** 10.1128/AAC.00795-21

**Published:** 2021-10-18

**Authors:** Sibo Wang, Shan Wang, Weishan Wang, Yi Dai, Zhongpeng Qiu, Wei Ke, Minghao Geng, Jing Li, Ke Li, Qingyuan Ma, Feng Li

**Affiliations:** a Department of Orthopedics, Tongji Hospital, Tongji Medical College, Huazhong University of Science and Technology, Wuhan, China; b Laboratory of Translational Medicine, School of Medicine, Shihezi University, Shihezi, Xinjiang, China

**Keywords:** ABZ-CS-MPs, IMRT, antiechinococcosis, *in vivo* imaging, spinal echinococcosis

## Abstract

Currently, there is a lack of clinically safe and effective treatment for spinal cystic echinococcosis (CE). Recent studies have shown that albendazole chitosan microspheres (ABZ-CS-MPs) and irradiation have certain anti-abdominal echinococcosis ability, so this study aims to compare the *in vitro* and *in vivo* therapeutic effects of ABZ-CS-MPs, intensity-modulated radiation therapy (IMRT), and combination therapy on spinal echinococcosis. First, protoscoleces were processed by different treatments to evaluate their respective antiechinococcosis effects by monitoring the viability change of protoscoleces. Then, the apoptotic status of protoscoleces was evaluated by detecting the changes of mitochondrial membrane potential, the expression of apoptosis proteins, and the ultrastructural alterations of protoscoleces. After that, we constructed a gerbil model of spinal CE and further applied B-ultrasound and magnetic resonance imaging (MRI) technology to assess the size of hydatid *in vivo*. Finally, the cysts were obtained and weighed to compare the inhibition rate in different groups. The combined therapy increased protoscoleces mortality to over 90% after 18 days, which showed the highest scolicidal effect. Moreover, confocal imaging, expression of apoptotic proteins, and ultrastructural changes of protoscoleces showed the highest apoptotic rate in this group. *In vivo*, the combination treatment also exhibited the highest cyst inhibition rate (61.4%). In conclusion, our results showed that ABZ-CS-MPs combined with IMRT could be a new treatment option for spinal CE. We also provided a method to evaluate the growth and metastasis of hydatid in animals with B-ultrasound and MRI technologies.

## INTRODUCTION

Echinococcus granulosus is a group of cestode tapeworms that act as the causative agents of cystic echinococcosis (CE), one of the primary neglected chronic diseases recently considered by the World Health Organization (1). As one of the three major tapeworms and due to its wide geographical distribution, the disease represents a significant public health problem and exerts a huge impact on domestic livestock (2, 3). The incidence rate of bone hydatid disease is low, accounting for only 0.2% to 4% of echinococcosis (4). The spine is one of the common parasitic sites of osteoechinococcosis, and the biological behavior is more complicated than that of liver and lung hydatid (5, 6). Patients with spinal echinococcosis usually have atypical clinical symptoms. *Echinococcus granulosus* is parasitic inside or outside the spinal cord cavity with low growth, causing bone destruction and pathological fracture (7). The skin above the spine can be broken, forming a long-lasting nonhealing fistula with effusion of discharge pus and hydatid debris. Symptoms and signs will gradually progress due to nerve compression (8), and paraplegia may eventually develop.

Currently, surgery is still the preferred option for the treatment of spinal echinococcosis. With the improvement of surgical methods and skills, the clinical therapeutic effects of hydatidosis have been dramatically improved. However, operation on lesions next to the nerve tissue still has significant risks. Some patients may experience severe anaphylactic shock or death due to the release of fluid in the cyst after surgery, which also leads to a high recrudescence rate of 25% (9). Chemotherapy with benzimidazole carbamates, such as albendazole (ABZ) and mebendazole (MBZ), is a conservative option in inoperable cases of spinal CE. Nevertheless, approximately 20 to 40% of patients respond poorly to chemotherapy (10), particularly those suffering from bone hydatid disease. The efficiency of drugs is hindered due to its shortcomings, including low absorption into blood circulation, poor solubility in water, as well as low permeation to the parasite cysts (11, 12). Moreover, subsequent side effects, including abnormal liver function, leucopenia, and alopecia have also been observed, which often result from high-dose administration with extended periods. Therefore, further research is still in need to explore novel therapeutic strategies to cure spinal CE, including combination treatments that are safer, more efficient, and noninvasive.

Previous studies have successfully synthesized albendazole chitosan microspheres (ABZ-CS-MPs) with good bio-adhesive properties, biodegradable ability, and low toxicity (13). Compared with the traditional albendazole, ABZ-CS-MPs significantly increases the blood concentration of the drug and is more effective in destroying and penetrating the germinal layer and cuticle of *Echinococcus*. These newly designed microspheres also have higher antiechinococcal efficacy *in vitro*, as they reduce parasite burden to a lower level compared to albendazole (14). Additionally, radiotherapy has also been investigated as another alternative treatment to inhibit the metacestodes of *E. granulosa*. The safe dose of X-ray widely used in the treatment of various tumors also shows parasiticidal effects and can modulate the phenotype and gene expression of the metacestodes (15, 16). Nevertheless, conventional radiotherapy is harmful to normal tissues. Intensity-modulated radiation therapy (IMRT), as the mainstream technology of radiotherapy, is suitable for most tumors and has been well evaluated in the clinical treatment of vertebral tumors (17, 18). Notably, IMRT is also a kind of high-precision radiotherapy concentrating high-dose radiation on the targeted area by using computed tomography (CT) images to reconstruct the three-dimensional tumor structure, which helps to reduce the dose to normal tissue around the lesion. These advances prompted us to test whether ABZ-CS-MPs or IMRT is possible in the treatment of spinal CE, and we hypothesized that ABZ-CS-MPs combined with IMRT is more effective than single therapy in the treatment of spinal echinococcosis.

Given the lack of proven spinal echinococcosis mouse models in the current research, we first constructed a stable spinal CE-infected mouse model. Then, we explored a method to dynamically monitor the growth and metastasis of echinococcosis *in vivo* by the application of B-ultrasound and magnetic resonance imaging (MRI). The main aim of this study is to evaluate the efficacy of ABZ-CS-MPs, IMRT, and a combination of the two therapies against spinal *E. granulosus in vitro* and *in vivo*.

## RESULTS

### ABZ-CS-MPs combined with IMRT exhibit potent parasiticidal activity against *E. granulosus* larval stages.

**FIG 1:**
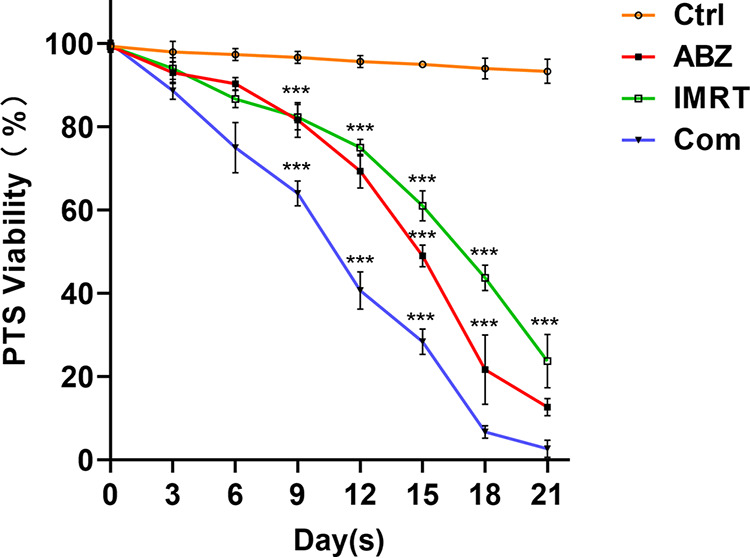
*In vitro* effect of ABZ-CS-MPs (ABZ), IMRT, and their combination (Com) on viability of protoscoleces (PTS) of *E. granulosus*. *E. granulosus* protoscoleces were cultured for 21 days *in vitro* in the presence of ABZ-CS-MPs (15 μM), IMRT (30 Gy), and ABZ-CS-MPs+IMRT; parasites incubated in culture medium containing DMSO served as controls (Ctrl). Data are the mean ± SD of three independent experiments. ***, Statistically significant difference (*P* < 0.001) compared with control.

To explore the effects of ABZ-CS-MPs combined with IMRT on the viability of CE cultured *in vitro*, the mortality of different groups of the protoscoleces was analyzed. As shown in Fig. 1, the number of dead protoscoleces increased with the exposure time of ABZ-CS-MPs (15 μM), and the viability decreased to 69.3% after 12 days of culture. In contrast, a notable loss of protoscoleces viability subject to IMRT (30 Gy) was observed after 15 days, with a 39% reduction in the number of viable protoscoleces. Intriguingly, a strong protoscolicidal effect was found in the combination group. In this case, protoscoleces mortality increased to over 90% after 18 days of incubation compared to 60% in the ABZ-CS-MPs group and 56.3% in the IMRT group (*P* < 0.001). In addition, there was no significant difference in antihydatid effect between the ABZ-CS-MPs and IMRT treatment groups in prescribed dose and radiation intensity (*P* > 0.05). Untreated protoscoleces remained at least 95 ± 1.0% viable during the complete assays.

### Accelerated apoptosis of protoscoleces exposed to ABZ-CS-MPs combined with IMRT.

**FIG 2:**
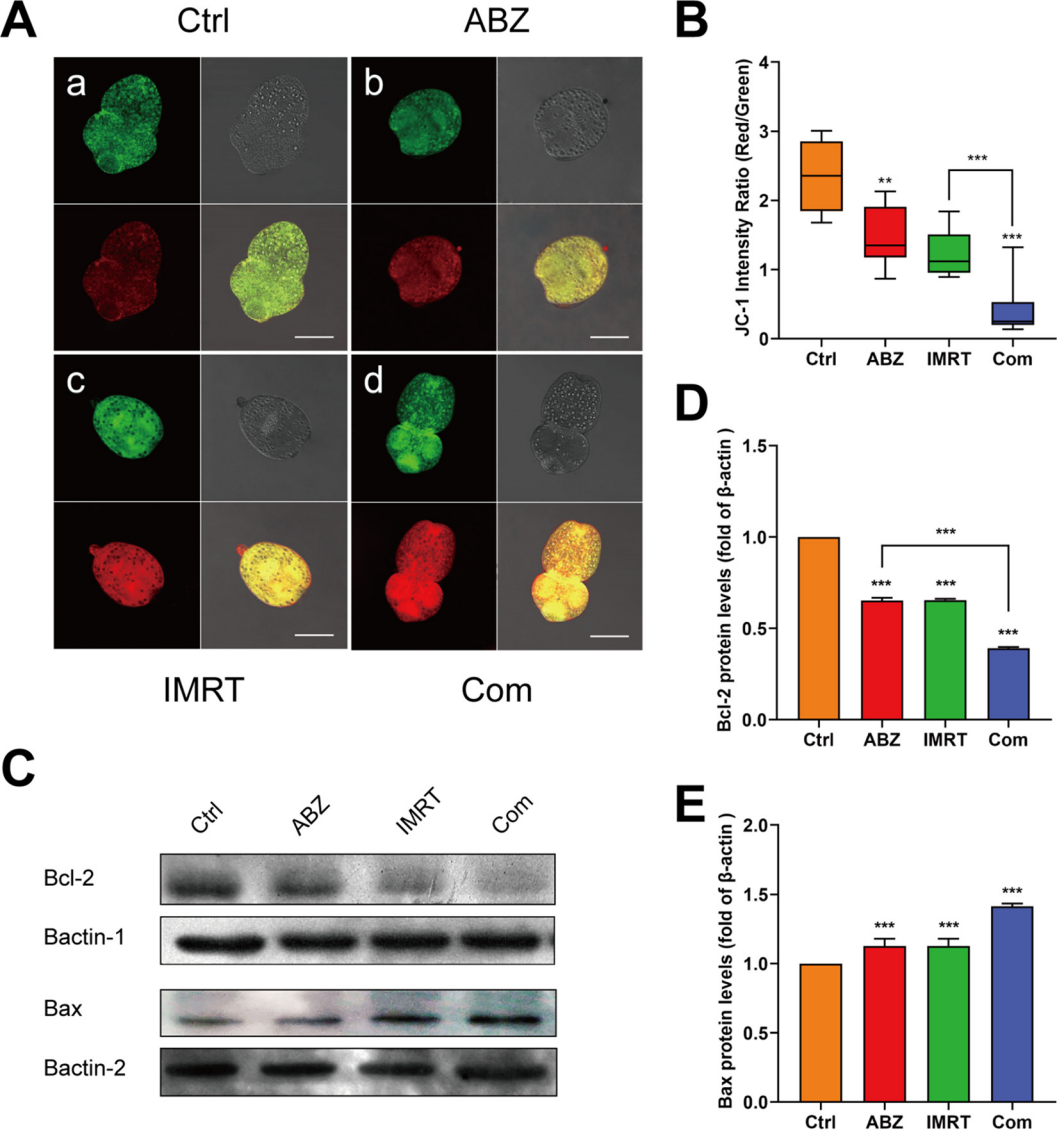
ABZ-CS-MPs- and IMRT-induced apoptosis of protoscoleces. Four groups of protoscoleces were incubated with JC-1 dye. Images were collected using confocal microscopy, and fluorescence was quantified. Representative images are shown. (A) Fluorescence images of different groups. Bars indicate 200 μm. (B) Red/green fluorescence ratios measured in the control and treatment group protoscoleces by Image J software. **, *P* < 0.01. The scale bar indicates 200 μm. (C, D, E) The expression of Bcl-2 and Bax in protoscoleces was evaluated by Western blotting analysis. Data are presented as mean ± SD of 3 independent experiments. **, *P* < 0.01; ***, *P* < 0.001.

To evaluated the antiechinococcal activity of the three treatment options, we used the ΔCm (mitochondrial membrane potential) indicator JC-1 to monitor the status of apoptosis in *E. granulosus* protoscoleces. JC-1 fluorescence of protoscoleces from the control, ABZ-CS-MPs, IMRT, and combination groups were analyzed by confocal microscopy. Following 48 h of treatment, untreated protoscoleces presented a ratio of red to green fluorescence with a mean value of 2.36 (Fig. 2Aa and Fig. 2B). In contrast, ABZ-CS-MPs-treated protoscoleces showed a lower mean ratio of around 1.48 (Fig. 2Ab and Fig. 2B). As for the IMRT group, the ratio of red to green fluorescence showed a further reduction, with a mean value of 1.24 (Fig. 2Ac and Fig. 2B). Obviously, the combination treatment resulted in the lowest ratio to 0.42 (Fig. 2Ad and Fig 2B) (*P* < 0.001), indicating that the combined treatment led to the most severe apoptosis in protoscoleces.

To detect the apoptosis of protoscoleces under different treatments, we examined the levels of Bcl-2 and Bax, the two typical apoptotic proteins. The results are shown in Fig. 2. The expression of Bcl-2 in the ABZ-CS-MPs, IMRT, and ABZ-CS-MPs+IMRT groups were significantly different from that of the control group (*P* < 0.001) (Fig. 2C and D). Furthermore, multiple comparisons suggested that the level of Bcl-2 was further decreased in the combination group compared with the ABZ-CS-MPs group alone (Fig. 2D). As for Bax protein, the three treatments showed a significant difference compared with the control group, but no difference was found among them (*P* > 0.05) (Fig. 2E).

### *In vitro* effect of the ABZ-CS-MPs combined with IMRT on ultrastructural characteristics of *E. granulosus* larval stage.

**FIG 3:**
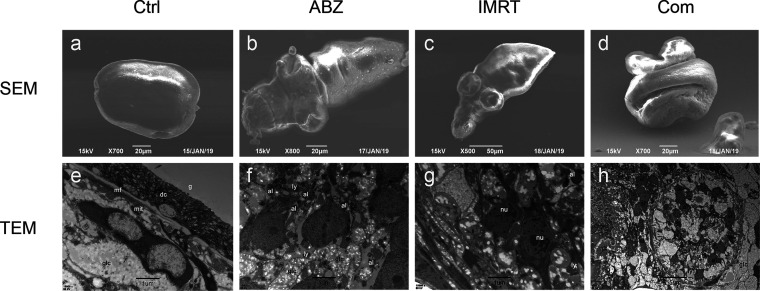
Ultrastructural changes of protoscoleces detected by SEM and TEM after ABZ-CS-MPs and IMRT treatment. Protoscoleces were tested after 48 h treatment. Protoscoleces in the ABZ-CS-MPs (15 μM) and IMRT (30 Gy) groups presented some contraction of partial region and shedding of microtriches and exhibited remarkable contraction and a scolex region showing loss of hooks in combined treatment in SEM. The control protoscoleces exhibited no ultrastructural alterations in parasite tissue. Bars indicate 50 μm in panel c and 20 μm in panels a, b, and d. In TEM, protoscoleces in the ABZ-CS-MPs+IMRT group showed an abundant amount of autophagosomes and lysosomes with nucleolysis (h), a distorted tegument and several autophagosomes appeared in the IMRT group (g), and some lysosomes and a reduction in glycogen storage were found in the ABZ-CS-MPs group (f). The ultrastructure in control protoscoleces appeared unaltered (e). glc, glycogen storage; mit, mitochondrion; g, glycocalyx; dc, distal cytoplasm; mf, muscular fibers; nu, nucleus; ld, lipid drop; ly, lysosomes; al, autophagolysosomes. Bars indicate 1 μm in panels e, f, and g and 5 μm in panel h.

Under scanning electron microscopy (SEM), no ultrastructural alterations in parasite tissue were exhibited in the control protoscoleces (Fig. 3a), while the ABZ-CS-MPs- and IMRT-treated protoscoleces showed some shedding of microtriches and contraction of partial region (Fig. 3b and c). Notably, the protoscoleces in the combined treatment group exhibited a great contraction and scolex region, showing loss of hooks (Fig. 3d). Moreover, from transmission electron microscopy (TEM) analysis, the tegument ultrastructure and its associated glycocalyx in the control group appeared to be unaltered (Fig. 3e), while a distorted tegument and several autophagosomes appeared in the germinal layer in the IMRT group (Fig. 3f). Some lysosomes and a reduction in glycogen storage were also found in the ABZ-CS-MPs-treated protoscoleces (Fig. 3g). Additionally, protoscoleces treated with the drug combined with X-ray presented an abundant amount of autophagosomes and lysosomes with nucleolytic and intracellular structure disorders (Fig. 3h).

### Significant effects of ABZ-CS-MPs combined with IMRT in the treatment of infected mice.

**FIG 4:**
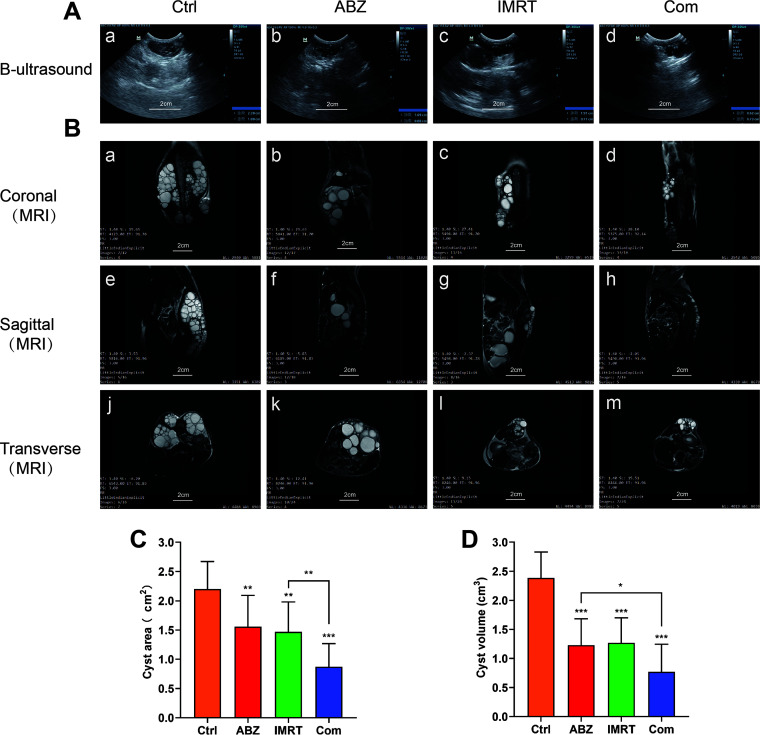
*In vivo* effect of combination of ABZ-CS-MPs with IMRT in a spinal CE model. After 6 weeks of infection, MRI and B-ultrasound were used to dynamically detect the size and distribution of cysts in gerbils. (A) The location, shape, boundary, and internal echo of abnormal echo focus, blood flow signal, as well as maximum lesion diameter were recorded. Clear image was reserved to calculate the lesion area (cm^2^). Bars indicate 2 cm. (B) The maximum diameter of the vesicles was measured, and the cross-sectional area of the vesicles was calculated and compared. ***, *P* < 0.001. (C) Images of sagittal, coronal plane, and transverse section were captured. MR diffusion software was used to analyze the images. Bars indicate 2 cm. (D) The maximum diameter was measured, and the volumes of cysts were calculated and compared. **, *P* < 0.01; ***, *P* < 0.001.

To explore the inhibitory effect of ABZ-CS-MPs, IMRT, and the combination of the two on E. *granulosus in vivo*, we constructed the spinal echinococcosis gerbil model. Protoscoleces were implanted in the paraspinal soft tissue of randomly assigned gerbils. After 6 weeks of infection, more than 70% of gerbils were successfully inoculated by B-ultrasound. MRI and B-ultrasound were used to detect the size and distribution of cysts in gerbils dynamically. Measurement of cyst area at the end of the 20-week treatment period showed that there was significant parasiticidal activity of ABZ-CS-MPs (1.56 ± 0.51cm^2^) or IMRT (1.47 ± 0.53cm^2^) treatment compared with that of the nontreated control group (2.20 ± 0.47 cm^2^) (Fig. 4A and C) (*P* < 0.01). Additionally, the combined treatment group (0.87 ± 0.39 cm^2^) presented the smallest cyst area compared to that of the other three groups (*P* < 0.01) (Fig. 4A and C). Moreover, similar results can be obtained from MRI in detecting the volume of cysts. The combined treatment group (0.77 ± 0.48 cm^3^) showed the highest effect in reducing parasite burden in comparison with that of mice treated with ABZ-CS-MPs (1.23 ± 0.46 cm^3^), treated with IMRT alone (1.27 ± 0.43 cm^3^), and those in the control group (2.38 ± 0.45 cm^3^) (*P* < 0.001) (Fig. 4B and D).

### Wet weight and inhibition rate of cysts.

**FIG 5:**
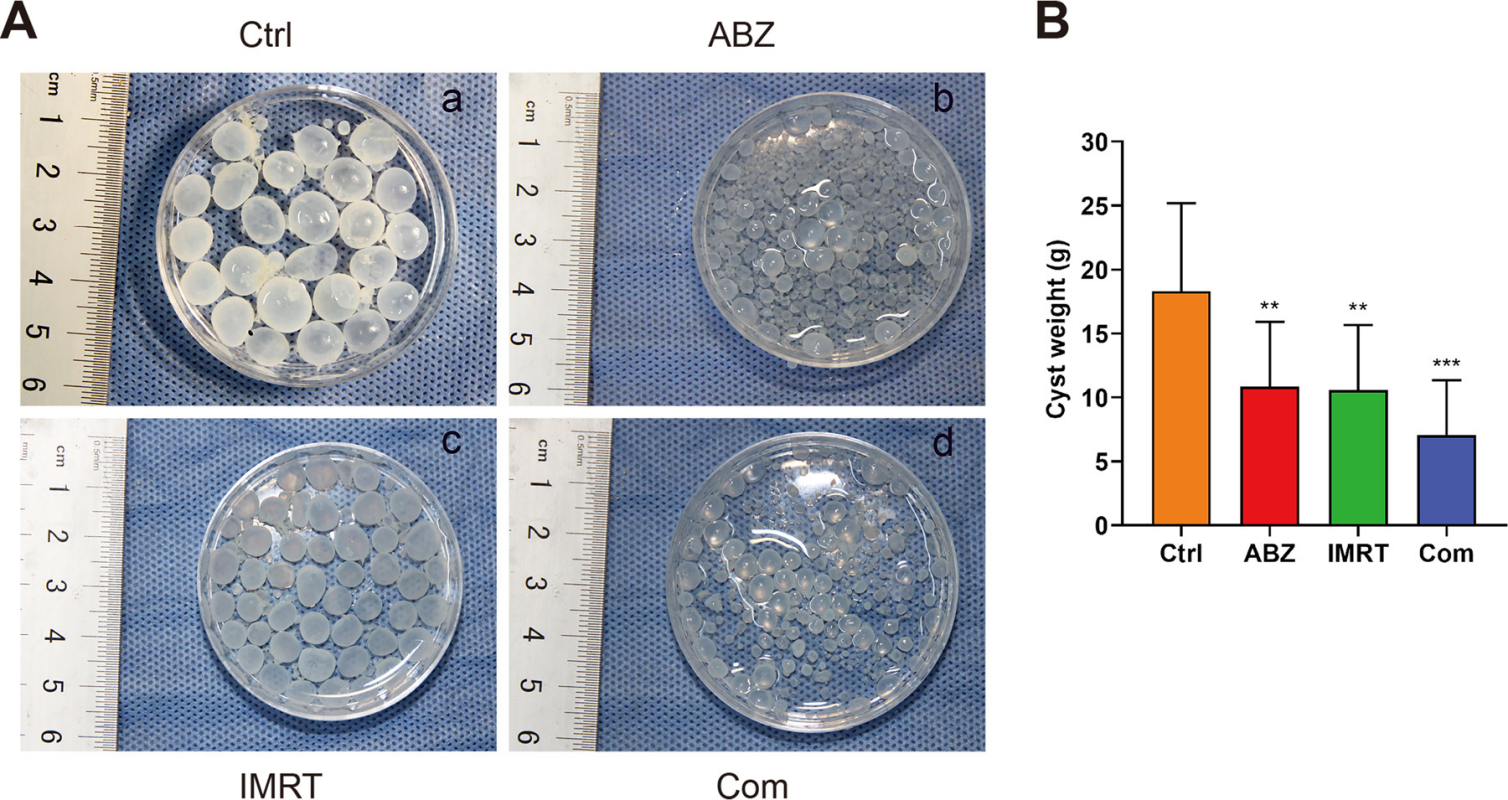
The wet weights of cysts in different treatment group. (A) After treatment, all cysts in different treated groups were taken out and the whole wet weight was measured. (B) Statistically significant difference compared with the control group; **, *P* < 0.01; ***, *P* < 0.001.

The weights of cysts (mean ± standard deviation [SD]) recorded in different treatments are summarized in Fig. 5. There were significant decreases in the cyst weights in mice treated with ABZ-CS-MPs (10.86 ± 5.06 g) (Fig. 5Ab and Fig. 5B) or IMRT (10.58 ± 5.08 g) (Fig. 5Ac and Fig. 5B) (*P* < 0.01) compared with those of mice in the untreated group (18.34 ± 6.86 g) (Fig. 5Aa and Fig. 5B). What is more, the gerbils in the combined treatment group had the smallest cyst weight around the spine (7.08 ± 4.29 g) (Fig. 5Ad and Fig. 5B), indicating a significant reduction in parasite burden (*P* < 0.001). Additionally, the gerbils treated with ABZ-CS-MPs+IMRT exhibited a 61.4% inhibition rate of the cyst, which was higher than that of ABZ-CS-MPs- (40.8%) and IMRT-treated (42.1%) mice.

### Toxicity of ABZ-CS-MPs+IMRT *in vitro* and *in vivo*.

**FIG 6:**
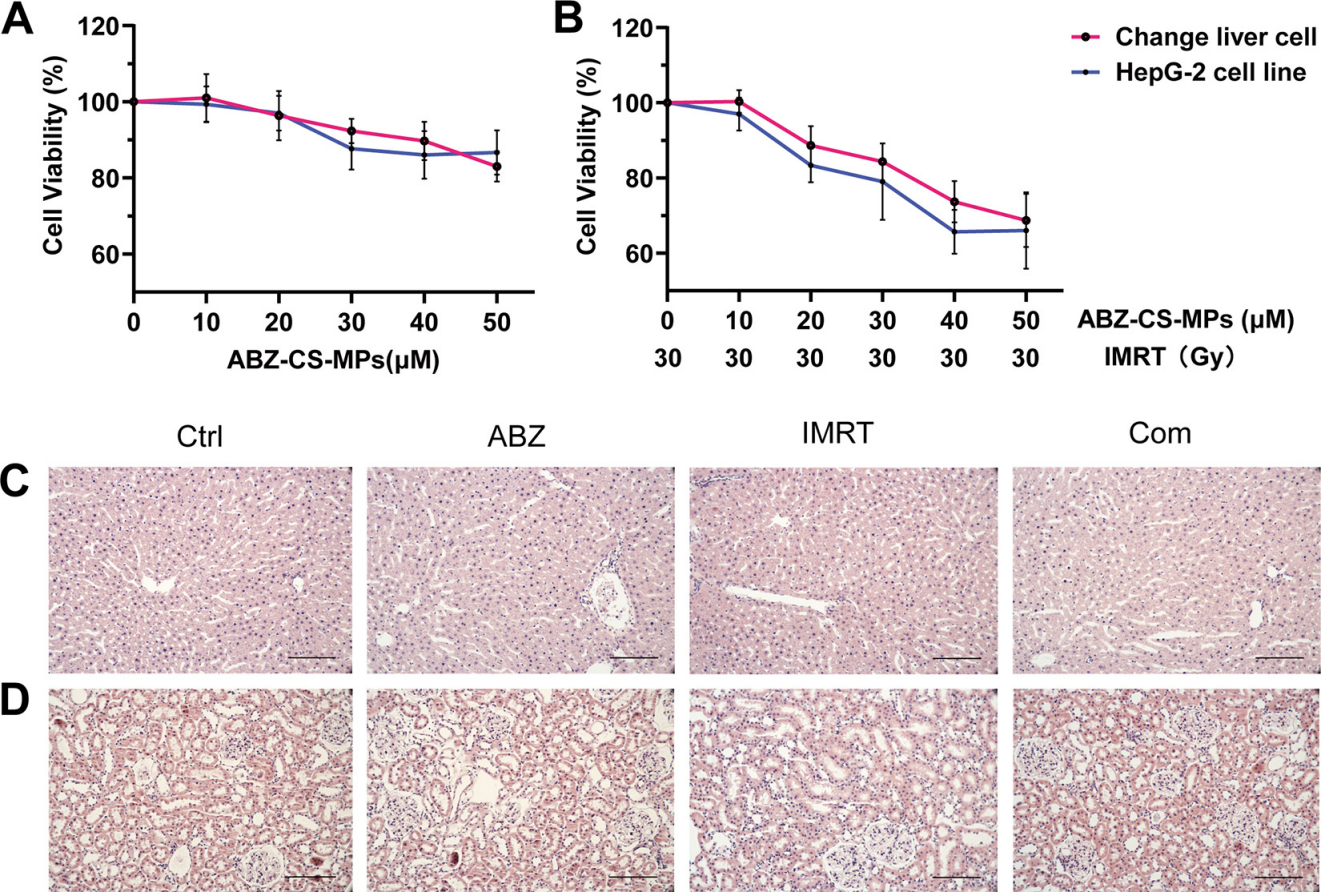
Toxicity *in vitro* and *in vivo*. (A, B) MTT assay was carried out to assess the cytotoxicity of single ABZ-CS-MPs and its combination with IMRT via the Chang liver cell line and HepG-2 cell line. H&E-stained images of liver (C) and kidney (D) tissues. Bars indicate 20 μm.

The efficacy and safety of single ABZ-CS-MPs or IMRT have been verified before (13, 19). In this study, lower dose and radiation intensity were selected and 3-(4,5-dimethyl-2-thiazolyl)-2,5-diphenyl-2H-tetrazolium bromide (MTT) assay was performed to further test the safety of this combination therapy. The cytotoxicity of single ABZ-CS-MPs and combination therapy were assessed using the MTT assay *in vitro* (Fig. 6). After being cultured in the solution with an ABZ-CS-MPs concentration of 20 μM, the viability of the normal cell line of Chang liver cells was higher than 90%, and that of the cancer cell line of HepG2 cells was higher than 95% with the same concentration (Fig. 6A). The viabilities of these two cell lines after combined treatment with ABZ-CS-MPs (20 μM) and IMRT (30GY) were 88.7% and 88.3% (Fig. 6B). The toxicity of combination therapy was assessed by morphological observation of liver and kidney tissues *in vivo*. Hematoxylin and eosin (H&E)-stained images revealed no obvious pathological changes and injuries in liver and kidney tissues (Fig. 6C and D). Moreover, no manifestations of central nervous system damage in the model were observed in the evaluation during the treatment period.

## DISCUSSION

Research on novel compounds for the treatment of CE requires a high investment in time and money. Therefore, a drug reuse strategy provides an alternative way to significantly shorten the traditional drug development process (20). Albendazole, the most common antiechinococcal drug, has always been criticized for its poor water solubility and low local blood concentration, let alone the various side effects caused by long-term use in clinical practice (21, 22). In our previous work, CS-MPs was encapsulated in liposomes as carriers of ABZ and showed a promising capacity against alveolar echinococcosis (AE) (13). In our study, the efficacy of ABZ-CS-MPs against CE was tested *in vivo* in a spinal gerbil model.

Radiotherapy, owing to its noninvasive and precise features, plays an increasingly important role in oncotherapy (23). Previously, extensive studies have shown X-rays have killing effects on the cultured protoscolex and cysts *in vitro* (17). One exciting aspect of this study is that we carried out IMRT in our *in vivo* and *in vitro* experiments, which improves the accuracy of the implementation process of the radiotherapy program and maximizes the irradiation of CE and, therefore, best protects the normal tissues around the cysts (24). A latest study has shown that IMRT (60 Gy) has the same antiechinococcus effect as conventional radiotherapy *in vivo*, and little damage was found to the sciatic nerve, fully demonstrating its validity and safety (19).

In our *in vitro* experiments, we tested the antiechinococcosis effects of ABZ-CS-MPs, IMRT, and combination therapy on *E. granulosus* larvae by monitoring the activity of protoscoleces. We confirmed that the combined treatment exhibits the most promising activity against CE compared with that of the ABZ-CS-MPs, IMRT, and control groups. However, in addition to the two states of survival and death, a brief and straightforward test is needed to verify the parasite's apoptosis at a time point. JC-1 dye, as a fluorescent probe for monitoring mitochondrial ΔCm, has been used in protoscolex to assess mitochondrial function (25, 26). Here, we tested the effects of ABZ-CS-MPs, IMRT alone, or the combination of them on *E. granulosus* larvae to determine the antiechinococcal impact in terms of the relative values of red/green fluorescence. In different treatment groups, the combination group represents the lowest red/green fluorescence ratio (Fig. 2B), indicating significant depolarization of mitochondria and apparent changes in cell membrane potential. The pharmacological action of ABZ-CS-MPs was enhanced after combination with IMRT in inhibiting the glucose metabolism of protoscolex. In order to further detect the damage caused by different treatments, we measured the expression of Bcl-2 and Bax proteins. Bcl-2 (also known as antiapoptotic protein) and its related cytoplasmic proteins are the critical regulators of cell apoptosis. The cell suicide program is essential for development, tissue homeostasis, and resistance to pathogens (27). Bax, known as a water-soluble protein homologous to Bcl-2, is an apoptosis promoting protein in the Bcl-2 protein family. Overexpression of Bax can antagonize the protective effect of Bcl-2 and turn on programmed cell death (28, 29). Our results showed that the expression of Bcl-2 in the ABZ-CS-MPs+IMRT group was the lowest compared with that of the control and single treatment group, while the expression of Bax was the highest among the four groups, indicating that the damage provoked by the combination is more substantial and appears to be broader than that observed with the drugs or irradiation alone. As far as we know, only a few studies have been carried out to test related apoptotic protein to investigate the efficiency of treatment on the management of CE. However, further studies may be necessary to confirm specific mechanisms of apoptosis induced by treatments.

Furthermore, the ultrastructural changes reflecting the degenerative effect of the drugs and irradiation on the parasite are obtained through TEM and SEM. The alteration of morphology and intracellular structure of the protoscoleces induced by combined treatment indicated that ABZ-CS-MPs+IMRT combination had the most potent effect against the protoscoleces (Fig. 3d and h). One interesting aspect of this study is that in the radiotherapy group, the nucleus of protoscolex cells showed more obvious dissolution and fragmentation (Fig. 3g). Considering the intense penetration of radiation, DNA molecules are broken and cross-linked mainly by the direct action of radiation (30, 31). On this basis, we found drug intervention combined with X-ray exposure could efficiently destruct the typical structure and induce cell apoptosis of protoscoleces.

To study the therapeutic effect *in vivo*, we constructed a gerbil model of spinal CE by referring the basic principles of modeling as similarity, repeatability, reliability, and practicability. After in-depth understanding of the anatomical structure of the gerbil spine, the muscle tissue in the spinal region was designated as the implant site. Intraspinal injection was abandoned because of serious direct damage to the central nervous system. In order to determine the stability and repeatability of the model, the evaluation system was established, and related criteria are as follows: (i) whether the implant is successfully diagnosed by dynamic monitoring of imaging (B-ultrasound, MRI), (ii) daily observation of the activity of the model mice (claudication and abnormal gait may suggest the presence of nerve injury), and (iii) observation of pain reflex and evaluation of muscle strength of gerbil after mechanical and tenderness stimulation (if there is a decrease in the range of motion of the lower limbs or a slow response to stimulation, the spinal nerve may be damaged). The existence of criterion i alone or any combination with criterion i can determine success in constructing the model. Based on the above criteria, more than 76% of gerbils were confirmed to be infected with hydatid after imaging diagnosis and were defined as type 5 spinal CE with regard to the Dew/Braithwaite & Lees classification in clinical practice (32).

After successful modeling, oral application of ABZ-CS-MPs (150 mg/kg), a safe dose of IMRT (60 Gy), and combined intervention in mice infected with the larval stage were performed. Here, we found that the area and volume of hydatid cysts under combined treatment were the smallest (Fig. 4), proving that ABZ-CS-MPs+IMRT is significantly more effective than the monotherapy alone.

It must be highlighted that this is the first report to apply B-ultrasound and MRI imaging technologies to hydatid models, which can better monitor the growth, metastasis, and *in vivo* mechanisms of diseases in the context of physiologically authentic environments in a noninvasive manner. In past decades, researchers have routinely relied on a digressive experiment to determine parasite burdens in organs of interest (33). Although useful, many shortcomings cannot be avoided. First, the model animal must be executed at the time of detection, so that the dynamic biological behavior of the parasite cannot be monitored in the same model, which means that significant individual differences between models may be ignored (34). Additionally, the convincing data must be obtained by sacrificing more models in multiple time points. Second, the traditional methods rely on targeted tissue to examine; Diffuseness to unexpected anatomical sites may easily be missed. In our study, we found that hydatid tissue in some gerbils penetrated the dorsal peritoneum and entered into the abdominal cavity through the sagittal and transverse sectional MRI images (Fig. 4Bf and g). The noninvasive characteristic of imaging technology surmounts many of these drawbacks, which helps to achieve coherent and dynamic research *in vivo*. There is also a point to explain. For spinal echinococcosis, micro-CT is more suitable for monitoring *in vivo*. However, CT scanning will bring additional radiation errors and affect the experimental results. In the MRI, we did not find apparent intraspinal infiltration. Further studies are needed to explore the vertebral bone destruction and the mechanism of echinococcosis transferring into the spinal canal.

Besides, the wet weight in the ABZ-CS-MPs+IMRT treatment group is 7.28 ± 4.29 g (Fig. 5B), significantly lower than that in the ABZ-CS-MPs or IMRT group. The corresponding cyst inhibition rate is 61.40%, which is consistent with our results *in vitro*.

The MTT assay *in vitro* indicated the absence of obvious toxic effects of combination therapy (Fig. 6A and B). In addition, morphological observations of liver and kidney *in vivo* confirmed the low toxicity *in vivo* (Fig. 6C and D). What should be noted is that the treatment strategies selected in this study have a certain research basis and have been extensively used in clinical treatment. Therefore, we did not repeatedly calculate the 50% inhibiting concentration (IC_50_) and the combination index (CI) of treatment. CI is commonly used in two kinds of chemical drugs. This is a study that combines physical therapy and chemotherapy, and there is no interaction or influence between radiation and albendazole molecules.

In conclusion, we offer strong evidence that repurposing the application of ABZ-CS-MPs combined with IMRT could be used as a new treatment option for spinal CE. Moreover, we provided a method to construct, established the model construction evaluation system, and for the first time successfully applied B-ultrasound and MRI techniques to evaluate the growth and metastasis of hydatid in animals under therapeutic intervention. These findings will provide a new platform and monitoring method for the research of spinal hydatid and even hydatid disease.

## MATERIALS AND METHODS

### Chemicals.

Unless otherwise stated, all culture media were purchased from Gibco-BRL, while biochemical reagents were acquired from Sigma-Aldrich (St. Louis, MO, USA). JC-1 dye was supplied by Life Technologies (Grand Island, New York, USA). The ABZ-CS-MPs were synthesized by the pharmacy department of the First Affiliated Hospital of Shihezi University, Xinjiang (encapsulation efficiency 80.0%, drug loading 12.5%). The synthesis of ABZ-CS-MPs has been described previously (13).

### Ethics statement.

All protocols involving animals followed the guidelines for the care and use of laboratory animals (8th edition), and procedures were approved by the Animal Care and Use Committee of Shihezi University. Unnecessary animal suffering was avoided throughout the study.

### *In vitro* viability testing assays on the larval stage of *E. granulosus*.

Protoscoleces of hydatid cysts were acquired aseptically from naturally infected sheep at an abattoir in Changji of the Xinjiang Province, China. Viable and morphologically intact protoscoleces (*n* = 3,000) were washed several times with phosphate-buffered saline (PBS) and cultured using RPMI 1640 medium supplemented with 10% fetal calf serum and antibiotics (penicillin, streptomycin, and gentamicin at 100 μg/ml) in 24-well culture plates. Protoscoleces were divided into four groups as follows: the drug treatment group treated with 15 μM ABZ-CS-MPs, the radiotherapy group exposed to 30 Gy X-ray per day, the combined treatment group, and the control group. Parasites incubated in culture medium containing dimethyl sulfoxide (DMSO) were used as controls. The distance between protoscoleces and radiation source was 100 cm. The X-ray covered an area of 10 cm by 10 cm. The irradiation intensity was 30 Gy, a power that has been previously tested to repress *E. granulosus* and has been proven to be safe for humans (3). The viability assessment was performed by using trypan blue staining and was visualized at ×40 magnification for 21 days. The protoscoleces were taken for viability assessment; each treatment condition was repeated three times.

### Therapeutic effectiveness of ABZ-CS-MPs, IMRT, and their combination *in vitro*.

JC-1 is an ideal fluorescent probe widely used to detect ΔΨm (mitochondrial membrane potential) during early apoptosis. The relative ratio of red and green fluorescence has been used to evaluate the degree of mitochondrial depolarization and viability of protoscoleces (35). Before staining, protoscoleces in the four groups were cultured for 48 h after different treatments. After that, parasites were stained with 10 mg/ml JC-1 dye and were dissolved in DMSO for 30 min. Then, the protoscoleces were washed three times with 20 mM HEPES buffer and were transferred to a laser confocal culture dish (1,000 cells/dish). The representative images were taken with a confocal microscope (Nikon Eclipse C1 Plus). The intensities of green (excitation/emission wavelength = 485/538 nm) and red (excitation/emission wavelength = 485/590 nm) fluorescence were analyzed in 20 protoscoleces from 4 groups.

Polypeptides were separated on 10% polyacrylamide gel by SDS-PAGE, and then electroblotting was performed on a nitrocellulose membrane (HyBond C; Amersham, Argentina). Bax (1:1,000) and Bcl-2 (1:1,000) primary antibodies (Beverly, MA, USA) were diluted with 5% bovine serum albumin (BSA) solution. Bax (1:1,000), Bcl-2 (1:1,000), and actin (1:1,000) were diluted with 5% skimmed milk, and the dilution ratio of goat anti-rabbit secondary antibody was 1:20,000 (Proteintech Group, Wuhan, Hubei, China). The condition of first antibody immune reaction was 4°C overnight, and the second was at room temperature for 2 to 4 h after membrane washing. After the film was washed, it was developed and exposed in a dark room. The gray value was analyzed by Image J image software.

### Ultrastructural and morphological studies of CE protoscoleces.

In order to explore the ultrastructure of protoscoleces after 48 h in different treatment groups, scanning and transmission electron microscopy (SEM and TEM) were used, respectively. Protoscoleces were washed three times with PBS and were fixed with 4% glutaraldehyde. After dehydration with ethanol gradient and embedding in epon812 epoxy resin, 60-nm sections were acquired with an ultramicrotome. Last, the stained protoscoleces were observed under the SEM and TEM (JEOL 1230, Japan).

### Construction of spinal echinococcosis model.

Mongolian gerbil male mice (50 to 77 g) aged 6 to 8 weeks were used to construct the spinal echinococcosis model. Gerbils were anaesthetized with isoflurane (the anesthetic concentration was 4% to 5%, and the maintenance concentration was 1% to 3%). The hair on their back was shaved to expose the skin above the spine, and the injection area was disinfected with alcohol. A syringe containing 2 ml of protoscoleces (2,000/ml) was inserted obliquely along with the pubic symphysis plane from both sides along the latissimus dorsi muscle to the lower part of the trapezius muscle. The protoscolex suspension was injected, and the needle was withdrawn slowly. The animals were divided into four groups with 25 animals in each group, and the treatments were carried out 6 weeks after infection. Animals in each group received the following treatment schemes for 3 months: group 1 was administered with placebo (control group), group 2 with ABZ-CS-MPs via intragastric administration (150 mg/kg, three times a week), group 3 with IMRT (60 Gy, three times a week), and group 4 was subjected to ABZ-CS-MPs+IMRT combined treatment.

### *In vivo* dynamic imaging monitoring of echinococcosis.

Four months after infection, the gerbils in different groups were examined by MRI and B-ultrasound every 2 weeks until the vesicles were removed out of the body. Philips Achieva 3.0T TX superconducting magnetic resonance imaging was used to scan spinal hydatid cyst. For such analysis, gerbils were put in the prone position after inhalation anesthesia and were placed in small animal MRI fixed stent. Scanning was performed according to the sequence of array space-sensitive coding technology (ASSET) calibration scan, T2W1, T2 fat saturation, T1W1, and diffusion weighted imaging (DWI) examination. A spin echo-planar imaging (SE-EPI) sequence was used to copy the axial T2W1 localization image. Before scanning, the b value was set to 300 s/mm^2^, 500 s/mm^2^, and 700 s/mm^2^ for scanning, and the parameters were set as follows: repetition time (TR) ranged from 3,350 to 3,675 ms, echo time (TE) ranged from 80.2 to 90.4, slice thickness was 4 mm, layer spacing was 1.0 mm, field of view (FOV) was 10 mm by 10 mm and the matrix was 128 by 128, the matrix wheel was stimulated twice, and scanning layers were 8 to 10 layers. MR diffusion software was used to analyze the images. The maximum cyst diameter was measured, and the volume (vol = 4/3 πABC, cm^3^) was calculated.

A T6-VET was used for ultrasonic examination. The probe frequency was set at 7 to 12 mHz. The muscle and soft tissue examination conditions were carefully selected, and the indicators of the instrument were adjusted to the best state. From the fourth week after vaccination, gerbils in each group were randomly chosen for ultrasonic examination every 2 weeks. After inhalation anesthesia, weighing mixture was smeared on the gerbils’ backs, which was depilated and prepared. Multisection and omnidirectional ultrasound examinations were performed. The soft tissue around the spine of each gerbil was examined emphatically, and the discovery of abnormal signals was regarded as a sign of successful infection. The location, shape, boundary, and internal echo of abnormal echo focus, blood flow signal, as well as maximum lesion diameter, were recorded in detail. The clear image was reserved for calculating the lesion area (cm^2^).

### Efficacy of ABZ-CS-MPs and its combination with IMRT against E. *granulosus in vivo*.

Each gerbil was treated for 12 weeks after successful implantation was confirmed by B-ultrasound. Then, the gerbils were euthanized followed by incision of the back skin. After exposure, the cysts were removed, and the soft tissues attached to the cysts were cleaned. Finally, we weighed the cysts and calculated cyst inhibition rates of different groups.

### Toxicity assessment of combined therapy.

The Chang liver cells or HepG2 cells (180 μl) were inoculated into 96-well plates to reach a density of 1 × 10^4^ cells/well and were subsequently cultured for 24 h. Five serial concentrations (10, 20, 30, 40, and 50 μM) of ABZ-CS-MPs were used in the cell culture medium, and 0.1% DMSO was employed as a control. After the plate was incubated for 48 h, 20 μl of 3-(4,5-dimethyl-2-thiazolyl)-2,5-diphenyl-2H-tetrazolium bromide (MTT) was added into each well and cells cultured for 4 h. Then, supernatant was discarded, and the remaining formazan was dissolved in 150 μl of DMSO. An ELx800 microplate reader was used to read the absorbance rate at 490 nm optical density.

At the end of the study, liver and kidney tissues were collected after sacrificing the models. Then, the tissue was embedded in paraffin after being fixed in 10% neutral formaldehyde for 24 h. The samples were sectioned into 5 μm thickness and stained with hematoxylin and eosin (H&E) for histopathological examination.

### Statistical analysis.

All data within experiments were compared, and significance was determined using the Student’s *t* test or the nonparametric Kruskal-Wallis test, as appropriate. Data were analyzed by SPSS 26.00. A *P* value of <0.05 was considered to be statistically significant.
